# The dynamics of immune responses to *Mycobacterium tuberculosis* during different stages of natural infection: A longitudinal study among Greenlanders

**DOI:** 10.1371/journal.pone.0177906

**Published:** 2017-06-01

**Authors:** Sascha Wilk Michelsen, Bolette Soborg, Lars Jorge Diaz, Soren Tetens Hoff, Else Marie Agger, Anders Koch, Ida Rosenkrands, Jan Wohlfahrt, Mads Melbye

**Affiliations:** 1 Department of Epidemiology Research, Statens Serum Institut, Copenhagen, Denmark; 2 Department of Infectious Disease Immunology, Statens Serum Institut, Copenhagen, Denmark; 3 Department of Clinical Medicine, University of Copenhagen, Copenhagen, Denmark; 4 Department of Medicine, Stanford School of Medicine, Stanford, California, United States of America; Fundació Institut d’Investigació en Ciències de la Salut Germans Trias i Pujol, Universitat Autònoma de Barcelona, SPAIN

## Abstract

**Objective:**

Understanding human immunity to *Mycobacterium tuberculosis* (*Mtb*) during different stages of infection is important for development of an effective tuberculosis (TB) vaccine. We aimed to evaluate immunity to *Mtb* infection by measuring immune responses to selected *Mtb* antigens expressed during different stages of infection over time and to observe sustainability of immunity.

**Methods:**

In a cohort study comprising East Greenlanders aged 17–22 years (2012 to 2014) who had either; undetectable *Mtb* infection, ongoing or prior *Mtb* infection at enrolment, we measured immunity to 15 antigens over a one-year period. Quantiferon-TB Gold testing (QFT) defined *Mtb* infection status (undetected/detected). The eligible study population of East Greenlanders aged 17–22 years was identified from the entire population using the Civil Registration System. From the source population 65 participants were selected by stratified random sampling according to information on *Mtb* infection stage. Retrospective and prospective information on notified TB (including treatment) was obtained through the mandatory TB notification system and was used to characterise *Mtb* infection stage (ongoing/prior). Immunity to 15 antigens including two QFT antigens, PPD and 12 non-QFT antigens (representing early, constitutive and latent *Mtb* infection) was assessed by measuring immune responses using whole-blood antigen stimulation and interferon gamma measurement.

**Results:**

Of 65 participants, 54 were considered *Mtb*-infected. Immunity to *Mtb* infection fluctuated with high annual risk of conversion (range: 6–69%) and reversion (range: 5–95%). During follow-up, five (8%) participants were notified with TB; neither conversion nor reversion was associated with an increased risk of progressing to TB.

**Conclusions:**

Our findings suggest that human immunity to natural *Mtb* infection over time is versatile with fluctuations, resulting in high levels of conversion and reversion of immunity, thus human immunity to *Mtb* is much more dynamic than anticipated. The study findings suggest future use of longitudinal assessment of immune responses when searching for TB vaccine candidate antigens.

## Introduction

Tuberculosis (TB) will remain a major global health challenge, with an estimated two billion individuals infected, unless new preventive measures are identified [1,2].

*Mtb* infection is traditionally classified as either latent *Mtb* infection (LTBI) without clinical symptoms (90%) or as TB with clinical symptoms (10%) [3,4]. Similarly, it is believed that *Mtb* alters its gene expression profile during different stages of infection due to interaction with various human host defence mechanisms, consequently also causing variation in the antigen repertoire exposed to the human immune system [5–9].

Understanding human immunity to *Mtb* infection, including immunity to potential novel TB vaccine antigens, during different stages of infection and sustainability of immunity over time, is considered necessary to develop a novel TB vaccine capable of halting progression from *Mtb* infection to TB [5]. Currently, only a limited number of *Mtb* antigens have been exploited for use in novel TB vaccines [5,6,10,11], with ESAT-6, TB10.4, and Ag85A-B antigens represented in numerous vaccine candidates [12]. Furthermore, little is known about the change in immune response to *Mtb* antigens when infection occurs and before TB disease develops. Hence, there is a continued need for expanding knowledge regarding the clinical relevance of immunity to *Mtb* antigens for future use in vaccine development.

The *Mtb* genes known to be expressed during different stages of infection include: early stage antigens (e.g. Ag85B) found to be highly expressed in the early replicative stage of infection [5,13]; constitutively expressed antigens (e.g. ESAT-6/TB10.4/CFP10) believed to be expressed throughout all stages of infection [14]; and LTBI antigens believed to be expressed predominantly when *Mtb* is quiescent and in a slow or non-replicative stage [5,15]. To our knowledge, no previous studies have evaluated immunity to *Mtb* infection by measuring immune responses to *Mtb* antigens from all stages in the *Mtb* infection cycle and change over time.

Greenland has a high TB incidence and represents a unique setting for *Mtb*-antigen evaluation as it is most likely free of cross-reacting antigens derived from non-tuberculous mycobacteria (NTM), enabling an unbiased evaluation of host immunity [16].

The study aims to evaluate host immunity to *Mtb* infection among individuals in a TB-high-endemic region by measuring immune responses to potential TB vaccine antigens believed to be expressed by the bacterium during different stages of infection, and to observe sustainability of host immunity over time.

## Methods

### Setting, study design and study population

Greenland is an autonomous part of the Kingdom of Denmark, but governed by the Greenland Self-government. The majority of the population is Inuit (89%) [17]. The population has universal and free access to health care, including free TB treatment. The study was performed in the Eastern part of Greenland in the Tasiilaq region with 3,008 inhabitants (1 January 2013) [17]. The average TB incidence in the study setting was 440/100,000 inhabitants/year from 1982 to 2012 [18]. All live-born children and new residents in Greenland are assigned a unique personal identification number through the Civil Registration System (CRS) [19], allowing individual follow-up through all national registers and providing information on e.g. place and date of birth, sex, place of residence and TB notification. To be categorised as Inuit in the present study, both parents should be registered as being born in Greenland. Neonatal Bacillus Calmette-Guérin (BCG) vaccination has been a part of the Greenlandic childhood vaccination programme since 1955, but was discontinued in 1991 and subsequently re-introduced in 1997 due to nationwide policy changes [18,20]. We assume all individuals born in Greenland from January 1, 1991, to December 31, 1996, to be unvaccinated. The assumption is evaluated elsewhere [18].

This was an explorative cohort study and the study population comprised primarily BCG unvaccinated Greenlanders aged 17–22 years living in Tasiilaq in the years 2012–2013. *A priori*, the size of the study population was set at 65 individuals. Among the eligible population identified through the CRS (N = 206), adequate information on *Mtb* infection and treatment status was obtainable for 181 individuals. QuantiFERON^®^-TB Gold testing (QFT) defined *Mtb* infection status (undetected/detected). The eligible study participants were categorised into four groups: (1) *Mtb* infection undetectable by QFT (N = 94), (2) ongoing *Mtb* infection, non-treated (N = 25), (3) prior *Mtb* infection, treated for notified TB (N = 41), and (4) prior *Mtb* infection, treated with preventive monotherapy (N = 21). Participants were invited randomly within each of the groups, however with oversampling of individuals with *Mtb* infection, and with particular focus on including individuals with ongoing *Mtb* infection, anticipating immune responses to *Mtb* antigens in this group. One week in advance, the participants received a personal letter of invitation, study information, and a consent form. Participants were enrolled in October-November 2012 and April 2013, and followed-up with one or two subsequent assessments in April and/or September 2013. Study staff assessed the participants at the local hospital and blood samples were obtained at each assessment. All participants were simultaneously followed-up for TB through national registers.

### Assessment of TB, TB treatment status, QFT and definition of *Mtb* infection

Information on TB diagnosis and treatment was obtained from the Greenlandic TB notification system where cases, following WHO case definitions, are notified to the National Board of Health with mandatory notification since 1955. Subsequent TB was defined as TB diagnosed from study enrolment to end of follow-up. Information on preventive monotherapy and QFT results prior to enrolment was obtained from medical records and the national laboratory database; results originating from routine TB diagnostics, contact tracing and population screening. The Greenlandic treatment regimens, curative treatment (TB) and preventive monotherapy, follow WHO recommendations.

The Greenlandic healthcare system and the present study used the commercialised QFT to assess detectable *Mtb* infection. QFT is a standardised IGRA measuring T-cell-induced immune responses to the *Mtb* antigens ESAT-6, CFP10, and TB7.7 [14,21,22]. QFTs were analysed at Statens Serum Institut, Denmark (study QFTs) and at the Central Laboratory, Queen Ingrid’s Hospital, Nuuk (prior QFTs) following the instructions of the manufacturer [22].

Ongoing *Mtb* infection was defined as a positive QFT (prior testing or at enrolment) without having received treatment (curative or preventive). Prior *Mtb* infection was defined as either having notified TB prior to enrolment or having received preventive monotherapy prior to enrolment. At enrolment, *Mtb* infection stages were defined as: (1) *Mtb* infection undetectable by QFT, (2) ongoing *Mtb* infection, non-treated, and (3) prior *Mtb*-infection, treated. The category prior *Mtb* infection included both participants treated for notified TB and with preventive monotherapy. We compared groups by *Mtb* infection status (*Mtb* infection undetectable by QFT vs. *Mtb* infection) and among *Mtb*-infected by treatment status (prior *Mtb* infection vs. ongoing *Mtb* infection).

### *Mtb* antigens, laboratory analyses and definition of immune responses

#### Selected Mtb antigens

We evaluated immune responses to 15 antigens categorised as follows: early *Mtb* infection stage antigens (Rv0203, Rv0642, Rv1196), constitutively expressed antigens (ESAT-6, CFP10, Rv3614, Rv3849, Rv3865, Rv3872), LTBI antigens (Rv1284, Rv2031, Rv2244, Rv2659, Rv2660c) and *Mtb* complex antigens (PPD). Currently, only ESAT-6, CFP10 and PPD are available in commercial tests. ESAT-6 and CFP10 were categorised as QFT antigens. Rv0203, Rv0642, Rv1196, Rv3614, Rv3849, Rv3865, Rv3872, Rv1284, Rv2031, Rv2244, Rv2659, and Rv2660c were categorised as non-QFT antigens. The non-QFT antigens were selected in June 2012 using the TB database at http://www.tbdb.org/. The expression profiles of the individual antigens were compared with the expression profile of Ag85B, which is a signature antigen of early expression [13]. The correlation of the individual antigens with Ag85B subsequently designated the antigen groups described above. Additional information on the selected antigens are presented in Table A in S1 File.

#### Laboratory analyses

Immune responses to *Mtb* antigens were assessed using a 7-day interferon gamma release assay (IGRA) with whole blood (WB) antigen stimulation done on site in Greenland and subsequent quantification of T-cell-induced interferon gamma (IFNy) by enzyme-linked immunosorbent assay (ELISA) in Denmark. In brief, WB was incubated with antigens in final peptide or PPD concentration 5 μg/ml in 200 μL volumes for 7 days at 37°C in a humidified incubator at 5% CO_2_. All samples were done in triplicates. WB incubated without antigen (NIL) and WB incubated with phytohaemagglutin (PHA, 5 μg/ml) were used as negative and positive controls [16]. Antigens were prepared as peptide pools with characteristics presented in Table A in S1 File. Rv0642, Rv1196 and Rv2659 were divided into pools a, b, and/or c due to antigen size, achieving peptide pools with a maximum of 19 peptides (median = 13). In Greenland, samples were stored at -20°C before and during transport to Denmark; transportation took place within one month. In Denmark, samples were stored at -80°C until ELISA analysis. The laboratory analysis is described in detail elsewhere [16].

#### Definition of immune responses, conversion and reversion

Immune responses were calculated as medians (IFNy values, pg/ml) for triplicate wells. An immune response to *Mtb* antigens was defined as positive if IFNy was >19.5 pg/ml AND fulfilled the following criteria: a) response (after NIL (background) subtraction) >41.5 pg/ml OR b) the ratio (between response and NIL) >antigen-specific cut-points estimated by mixture models. Antigen conversion was defined as converting from a negative to a positive immune response; antigen reversion was defined as reverting from a positive to a negative immune response. Immune responses to antigen pools (Rv0642, Rv1196, Rv2659) or groups of antigens (QFT, constitutive, early, LTBI, Rv2659/Rv1196) were defined as having an immune response to at least one of the pools or antigens, and IFNy response magnitude was defined by the maximum response. Based on our findings we additionally evaluated two combinations of antigens: Rv2659/Rv1196 (as described above) and CFP10-Rv2659/Rv1196 requiring having an immune response to both CFP10 and Rv2659/Rv1196.

#### Antigen specific cut-points

The cohort of the 65 study participants were nested in a larger cohort study of 911 Greenlanders [16] and the definition of a positive immune response was derived from the sum of immunological data from this larger study population. In the cohort of 911 Greenlanders, 19.5 pg/ml was the observed 95% quantile of the NIL IFNy values and 41.5 pg/ml was the observed 99% quantile of the NIL IFNy values [16]. The antigen specific cut-points estimated by mixture models were based on the ratios between antigen stimulated wells and NIL wells for the 65 participants at first assessment, except for Rv1284, Rv2660c, Rv2659, Rv3849, and PPD. For Rv3849 the antigen specific cut-point was estimated using information from all three assessments, as estimation convergence could not be achieved by first assessment information only. For Rv1284, Rv2659, Rv2660c, and PPD the antigen specific cut-points were estimated using immunological data from the cohort of 911 Greenlanders described above. The logarithm to the ratios were calculated as log((IFNy in antigen stimulated wells+0.1)/(IFNy in NIL wells+0.1)), 0.1 was added IFNy responses on both sides of the fraction to allow for IFNy values of zero. The distribution of the logarithm to the ratio was modelled as a mixture of two normal distributions with the same variance but different means estimated in a mixture model by an EM-algorithm using the R package mixtool [23,24]. The two normal distributions were interpreted as the positive immune responses and the negative immune responses, allowing for estimation of sensitivity and specificity for a given cut-point. The estimation process is illustrated elsewhere [16]. In the analyses, the cut-point ratios were chosen so that the estimated specificity was 95%. The estimated cut-points used in the definition of a positive immune response in the analyses are presented in Table A in S1 File. The method is described in detail elsewhere [16].

### Statistical analysis

Homogeneity among participants with *Mtb* antigen immune responses (prior *Mtb* infection vs. ongoing *Mtb* infection) was evaluated for prevalence by logistic regression and for IFNy response by Kruskal-Wallis test. The annual risk of conversion was estimated from the conversion rate. The conversion rate was estimated among participants with a negative immune response at enrolment with at least two subsequent assessments using binomial regression with a complementary log-log link and with the logarithm of the observation time as offset. The annual risk of reversion was estimated in a similar way, except it was based on participants with a positive immune response at enrolment or at second assessment, with at least one subsequent assessment. Associations between *Mtb* infection groups and risk of conversion/reversion were evaluated using the above binomial regression. The average annual conversion risk for a group of antigens was estimated using the same model but with data for each antigen included in the analyses assuming similar conversion rates for all antigens. Associations between positive antigen immune responses and subsequent TB were evaluated by Hazard Ratios (HRs) using Cox regression with age as underlying time scale, with adjustment for *Mtb* infection status (undetectable/detectable) and with follow-up until the first of the following events: TB, death or end of follow-up (31 December 2014). In the analyses of immune responses at enrolment (negative/positive), all participants were included and followed from enrolment. In the analyses of conversion and reversion during follow-up (no/yes), all participants assessed at least twice were included; follow-up began at second assessment and exposure was allowed to be time-dependent. If only first and third assessments were available, follow-up began at the midpoint between first and third assessment with the assumption that any observed change between first and third assessment happened before beginning of follow-up. A substantial CPF10 and ESAT-6 increase was defined as >75% quantile of changes for all participants between first and second assessment. All tests and 95% confidence intervals (CIs) were based on Wald statistics. Analyses were performed using the R software.

### Ethical considerations

The study fulfilled the Helsinki Declaration II. Written and informed consent was given by all participants and by parents or legal guardians of children <age 18. Child acquiescence was required for children <age 18. All participants with a positive QFT were referred to the local hospital for further evaluation. In the Tasiilaq region, individuals with a positive QFT but without clinical indication of TB, were followed closely by the TB health personnel with clinical assessments. The Commission for Scientific Research in Greenland (approval No. 2012–4) and the Danish Data Protection Agency approved the studies.

## Results

Overall, 65 participants were enrolled (60 in October-November 2012 and 5 in April 2013). Table 1 and Table B in S1 File present characteristics at enrolment and the 65 participants were categorised as follows into one of three groups: 1) undetectable *Mtb* infection at enrolment as measured by QFT: 11 (17%), 2) ongoing *Mtb* infection: 22 (34%) and 3) prior *Mtb* infection: 32 (49%). The median age was 19 years and few participants were BCG-vaccinated N = 6 (9%). More women had received prior treatment as compared with men (61% vs. 34%) and more men developed TB during follow-up as compared with women (14% vs. 3%). Participation was not associated with sex or BCG vaccination (Table C in S1 File).

**Table 1:** Characteristics at enrolment by Mtb infection status among 65 young adults in East Greenland.

Characteristics at enrolment	All						All
		*Mtb* infection undetectable by QFT	*Mtb* infection				Subsequent TB
			All	Ongoing	Prior		N
				Non-treated	Treated with preventive monotherapy	Treated for notified TB	
	N	N	N	N	N	N	
**All**	65	11	54	22	12	20	5
**Sex**							
Women	36	5	31	9	8	14	1
Men	29	6	23	13	4	6	4
**Age (years)**							
16–19	43	8	34	11	8	15	4
20–22	22	3	18	11	2	5	1
**Ethnicity**							
Inuit	64	10	54	22	12	20	5
Non-Inuit	1	1	0	0	0	0	0
**BCG vaccination**							
No	58	10	48	19	12	17	4
Yes	6	0	6	3	0	3	1
Unknown	1	1	0	0	0	0	0
**Year of:** **prior positive QFT or TST/ notified TB**							
1996–2009	15	-	15	12	0	3	1
2010–2012	37	-	37	10	12	15	2
2012							
In TB treatment	3	-	-	-	-	3	-
None	11	11	0	0	0	0	2
**Clinical information**							
Pulmonary TB						16	4
Extra-pulmonary TB						4	1
**Assessed in**							
September 2012	60	11	49	18	12	19	5
April 2013	57	9	48	20	10	18	4
September 2013	57	10	47	20	9	18	5
**Number of times assessed**							
Two	60	10	50	20	11	19	5
Three	49	9	40	16	8	16	4

Subsequent TB: TB notified to the National TB register at any point from study enrolment to end of follow-up.

*Mtb* infection was defined a positive QFT (prior or at enrolment) and categorised as ongoing (non-treated) or prior (treated for notified TB or having received preventive monotherapy).

QFT: QuantiFERON^®^-TB Gold test. TST: Tuberculin skin test. BCG: Bacillus Calmette-Guérin vaccination status estimated from birth cohort.

For further categorisation of year of QFT or notified TB/preventive monotherapy among participants at enrolment, please see Table B in S1 File.

### Immunity at enrolment

**Fig 1:**
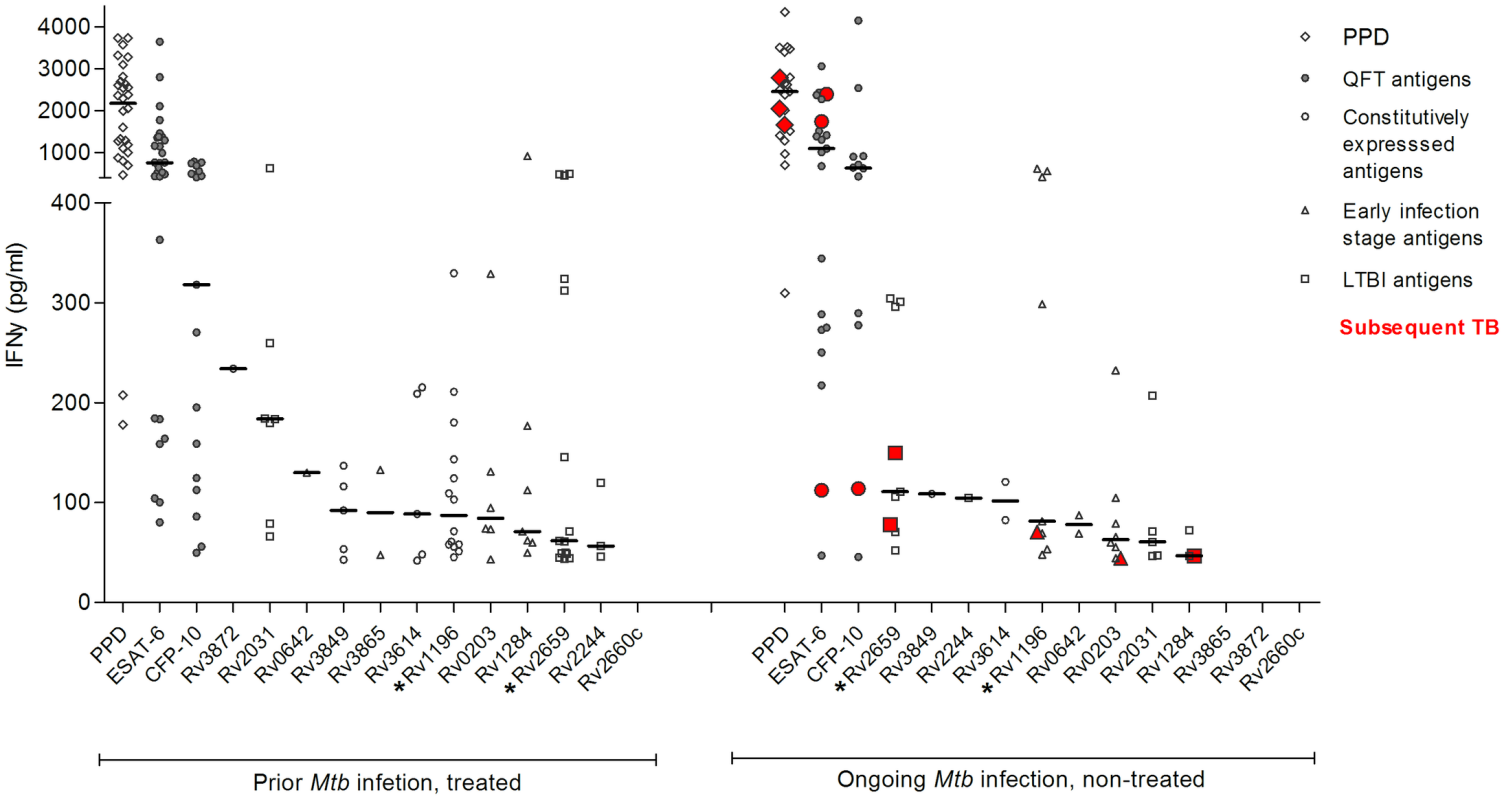
IFNy responses among participants with *Mtb* antigen immune responses at enrolment among participants with *Mtb* infection. Three participants (red) categorised at *Mtb* infected non-treated develop TB. *The two most prevalent non-QFT antigens in each group. All values are antigen responses after background subtraction in pg/ml with medians.

Among all 65 participants, 42 (65%) had an immune response to at least one of the 12 non-QFT antigens and 34% to three or more non-QFT antigens (Fig A in S1 File). Table 2 presents the prevalence of positive immune responses and the median IFNy response by *Mtb* infection stage at enrolment regardless of subsequent TB, reversion of immune responses, or QFT conversion during follow-up. The prevalence was highest for PPD and QFT antigens (range 45%-80%) and lowest for constitutively expressed antigens (range 2%-13%). Rv1196 and Rv2659 had the highest prevalence, while immune responses to Rv2660c were not detectable in the study population. Four participants without detectable *Mtb* infection at enrolment had immune responses to Rv2031, Rv2659, and Rv3849. Prevalence and IFNy response levels did not differ among participants with prior as compared with ongoing *Mtb* infection, except for the QFT IFNy response level, which was significantly higher for participants with ongoing *Mtb* infection (Table 2). For the distribution of IFNy responses at enrolment, see Fig 1; immune responses among participants with subsequent TB have been highlighted in red, all occur among participants with ongoing *Mtb* infection at enrolment.

**Table 2:** Prevalence of immune responses and median IFNy response among participants at enrolment by *Mtb* infection status, among 65 young adults in East Greenland.

	All			*Mtb* infection undetectable by QFT			*Mtb* infection									Prevalence: prior vs. ongoing P value^a^	IFNy response: prior vs. ongoing P value^b^
							All			Ongoing, non-treated			Prior, treated^h^				
	%	(N)	IFNy response median (range) pg/ml	%	(N)	IFNy response median (range) pg/ml	%	(N)	IFNy response median(range) pg/ml	%	(N)	IFNy response median (range) pg/ml	%	(N)	IFNy response median (range) pg/ml		
**All**		(65)			(11)			(54)			(22)			(32)			
**QFT**	80	(52)	209 (16–400)	0	(0)	-	96	(52)	210 (16–400)	95	(21)	400 (16–400)	97	(31)	157 (16–400)	*0*.*79*	***0*.*01***
**7-day IGRAs**																	
**PPD**^c^	80	(51)	2361 (178–4366)	0	(0)	-	94	(51)	2361 (178–4366)	95	(21)	2457 (310–4366)	94	(30)	2177 (178–3741)	*0*.*71*	*0*.*31*
**QFT Ags**^d^																	
**(any**^e^**/max**^f^**)**	**80**	**(52)**	**850 (47–4156)**	0	0	-	**96**	**(52)**	**850 (47–4156)**	**95**	**(21)**	**1099 (47–4156)**	**97**	**(31)**	**764 (80–3647)**	*0*.*79*	*0*.*24*
ESAT-6	78	(51)	764 (47–3647)	0	(0)	-	94	(51)	764 (47–3647)	95	(21)	1099 (47–3061	94	(30)	756 (80–3647)	*0*.*79*	*0*.*41*
CFP10	45	(29)	429 (46–4156)	0	(0)	-	54	(29)	429 (46–4156)	55	(12)	633 (46–4156)	53	(17)	318 (50–784)	*0*.*92*	*0*.*11*
**Constitutive**^g^																	
** (any/max)**	**17**	**(11)**	**109 (48–234)**	**18**	**(2)**	**51 (49–53)**	**17**	**(9)**	**121 (48–234)**	**14**	**(3)**	**109 (82–121)**	**17**	**(6)**	**171 (48–234)**	*0*.*62*	*0*.*30*
Rv3614	11	(7)	89 (42–215)	0	(0)	-	13	(7)	89 (42–215)	9	(2)	102 (82–121)	16	(5)	89 (42–215)	-	-
Rv3849^c^	13	(8)	73 (43–137)	18	(2)	51 (49–53)	11	(6)	100 (43–137)	5	(1)	109 (-)	16	(5)	92 (43–137)	-	-
Rv3865	3	(2)	90 (48–133)	0	(0)	-	4	(2)	90 (48–133)	0	(0)	-	6	(2)	90 (48–133)	-	-
Rv3872	2	(1)	234 (-)	0	(0)	-	2	(1)	234 (-)	0	(0)	-	3	(1)	234 (-)	-	-
** Early Ags**																	
** (any/max)**	**40**	**(26)**	**95 (44–617)**	**0**	**(0)**	-	**48**	**(26)**	**95 (44–617)**	**54**	**(12)**	**83 (44–617)**	**44**	**(14)**	**106 (46–329)**	*0*.*44*	*0*.*72*
Rv0203^c^	22	(14)	74 (43–329)	0	(0)	-	26	(14)	74 (43–329)	36	(8)	63 (44–232)	19	(6)	85 (43–329)	-	-
Rv0642	5	(3)	87 (69–130)	0	(0)	-	6	(3)	87 (69–130)	9	(2)	78 (69–87)	3	(1)	130 (-)	-	-
Rv1196	35	(23)	81 (45–617)	0	(0)	-	43	(23)	81 (45–618)	41	(9)	81 (48–618)	44	(14)	87 (45–329)	*0*.*84*	*0*.*38*
** LTBI Ags**																	
** (any/max)**	**55**	**(36)**	**105 (44–918)**	**36**	**(4)**	**61 (50–88)**	**60**	**(32)**	**128 (44–918)**	**60**	**(13)**	**106 (47–304)**	**60**	**(19)**	**177 (44–918)**	*0*.*98*	*0*.*60*
Rv2031^c^	20	(13)	79 (47–624)	9	(1)	50 (-)	22	(12)	129 (47–624)	23	(5)	61 (47–207)	22	(7)	184 (66–624)	-	-
Rv2244	6	(4)	80 (46–120)	0	(0)	-	7	(4)	80 (46–120)	5	(1)	104 (-)	9	(3)	57 (46–120)	-	-
Rv1284	15	(10)	67 (47–918)	0	(0)	-	19	(10)	67 (47–918)	14	(3)	47 (47–72)	22	(7)	71 (50–918)	-	-
Rv2659	43	(28)	74 (43–491)	36	(4)	61 (43–88)	44	(24)	92 (44–491)	41	(9)	111 (52–304)	47	(15)	62 (44–491)	*0*.*66*	*0*.*42*
Rv2660c	0	(0)	-	0	(0)	-	0	(0)	-	0	(0)	-	0	(0)	-		
** Combination of antigens**																	
Rv2659/Rv1196	55	(36)	97 (43–617)	36	(4)	61 (43–88)	30	(32)	110 (44–617)	54	(12)	130 (48–617)	63	(20)	90(44–491)	*0*.*56*	*0*.*24*

^a^ P values relate to homogeneity test for odds of having an immune response to the selected *Mtb* antigen among participants with *Mtb* infection; prior vs. ongoing.

^b^ P values relate to homogeneity for IFNy medians evaluated by the Kruskal-Wallis test among participants with *Mtb* infection; prior vs. ongoing.

^c^ One donor missing one stimulation.

^d^ Ags: antigens.

^e^ Immune responses for groups of antigens (QFT, constitutive, early, LTBI, Rv2659/Rv1196) were defined as having an immune response to at least one of the antigens and

^f^the IFNy response magnitude was defined by the maximum response.

^g^ Constitutive antigens.

^h^ For numbers on participants treated with preventive monotherapy and participants treated for notified TB, please see Table D in S1 File.

### Immunity over time and during different stages of infection

Of the 65 participants, 60 (92%) were assessed at least two times and 49 (75%) three times during 11 months (Table 1). Tables 3 and 4 present the estimated annual conversion and reversion risk during follow-up. The risks are cumulative risks, e.g. risk of conversion is the risk of ever converting, regardless of subsequent reversion, among participants who were without immune responses to *Mtb* antigens at enrolment. Overall, there was a high annual conversion and reversion risk for all non-QFT antigens; conversion (range 6–69%) and reversion (range 41–95%). For participants reverting from first to second assessment, the median IFNy level at second assessment was 7.4 pg/ml (Fig B in S1 File), thus the high reversion risk could not be ascribed to IFNy responses decreasing to levels just below the cut-point.

**Table 3:** Estimated annual conversion risk during follow-up among participants without detectable *Mtb* antigen immune responses at enrolment by *Mtb* infection status for 60 participants assessed more than once.

	All			*Mtb* infection undetectable by QFT				*Mtb* infection									
							Conversion risk: *Mtb* infection QFT un-detectable vs. detectable P value^a^	All			Ongoing, non-treated			Prior, treated^d^			Conversion risk QFT detectable *Mtb* infection, prior^d^ vs. ongoing P value^b^
		Without detectable *Mtb* antigen immune responses at enrolment			Without detectable *Mtb* antigen immune responses at enrolment				Without detectable *Mtb* antigen immune responses at enrolment			Without detectable *Mtb* antigen immune responses at enrolment			Without detectable *Mtb* antigen immune responses at enrolment		
	Estimated annual conversion risk	Convert during follow-up	Total	Estimated annual conversion risk	Convert during follow-up	Total		Estimated annual conversion risk	Convert during follow-up	Total	Estimated annual conversion risk	Convert during follow-up	Total	Estimated annual conversion risk	Convert during follow-up	Total	
	%	N	N	%	N	N		%	N	N	%	N	N	%	N	N	
**QFT**	**26**	3	12	**31**	3	10	*1*.*00*	**0**	0	2	**0**	0	1	**0**	0	1	-
**7-day IGRAs**																	
** PPD**	**54**	7	13	**50**	5	10	*0*.*57*	**67**	2	3	**77**	1	1	**59**	1	2	*0*.*74*
** QFT antigens**																	
Any^c^	**54**	7	12	**48**	5	10	*0*.*34*	**77**	2	2	**77**	1	1	**77**	1	1	*1*.*00*
ESAT-6	**58**	8	13	**48**	5	10	*0*.*14*	**86**	3	3	**77**	1	1	**91**	2	2	*0*.*70*
CFP10	**39**	12	32	**31**	3	10	*0*.*54*	**42**	9	22	**40**	3	8	**44**	6	14	*0*.*88*
** Constitutive antigens**																	
Any^c^	**30**	14	50	**36**	3	8	*0*.*64*	**28**	11	42	**45**	8	18	**14**	3	24	***0*.*048***
Rv3614	**24**	12	54	**30**	3	10	*0*.*61*	**23**	9	44	**34**	6	19	**14**	3	25	*0*.*14*
Rv3849	**15**	7	51	**25**	2	8	*0*.*38*	**13**	5	43	**28**	5	19	**0**	0	24	*1*.*00*
Rv3865	**6**	3	58	**11**	1	10	*0*.*50*	**5**	2	48	**6**	1	20	**4**	1	28	*0*.*81*
Rv3872	**10**	5	59	**0**	0	10	-	**12**	5	49	**17**	3	20	**8**	2	29	*0*.*38*
** Early antigens**																	
Any^c^	**51**	19	35	**30**	3	10	*0*.*16*	**59**	16	25	**71**	8	9	**50**	8	16	*0*.*24*
Rv0203	**25**	11	47	**21**	2	10	*0*.*73*	**27**	9	37	**51**	7	13	**10**	2	24	***0*.*02***
Rv0642	**14**	7	57	**21**	2	10	*0*.*46*	**12**	5	47	**6**	1	18	**16**	4	29	*0*.*39*
Rv1196	**53**	21	38	**31**	3	10	*0*.*15*	**60**	18	28	**70**	10	12	**50**	8	16	*0*.*23*
** LTBI antigens**																	
Any^c^	**71**	23	27	**61**	5	7	*0*.*45*	**75**	18	20	**77**	8	8	**73**	10	12	*0*.*82*
Rv2031	**34**	15	47	**12**	1	9	*0*.*20*	**39**	14	38	**61**	10	16	**20**	4	22	***0*.*02***
Rv2244	**14**	7	56	**0**	0	10	-	**17**	6	46	**18**	3	19	**17**	4	27	*0*.*93*
Rv1284	**13**	6	51	**20**	2	10	*0*.*44*	**11**	4	41	**12**	2	18	**10**	2	23	*0*.*84*
Rv2659	**69**	28	35	**61**	5	7	*0*.*60*	**71**	23	28	**68**	10	12	**73**	13	16	*0*.*76*
Rv2660c	**-**	0	60	**-**	0	10	-	**-**	0	50	**-**	0	20	**-**	0	30	-
**Combinations of antigens**^c^																	
Rv2659/Rv1196	**72**	24	27	**61**	5	7	*0*.*42*	**76**	19	20	**77**	9	9	**75**	10	11	*0*.*92*
CFP10 and Rv2659/Rv1196	**46**	19	41	**31**	3	10	*0*.*31*	**50**	16	31	**56**	7	12	**46**	9	19	*0*.*60*

The table presents three columns per group (e.g. all, *Mtb* infection undetectable by QFT, *Mtb* infection). The right column is the total number of participants without detectable *Mtb* antigen immune responses at enrolment for each antigen (e.g. for Rv0203, 47 participants did not have a detectable immune response to Rv0203 at enrolment). The middle column presents the number of participants who convert during follow-up (e.g. for Rv0203, 11 of 47 participants convert from a negative to a positive immune response to the antigen during follow-up), and the left column presents the estimated annual conversion risk (e.g. for Rv0203 the annual conversion risk is 25%).

The estimated annual conversion risk was based on all conversion events, regardless of subsequent reversion.

^a^ P values relate to homogeneity test for odds of conversion of *Mtb* antigen immune responses among all participants by *Mtb* infection status; QFT undetectable vs. detectable.

^b^ P values relate to homogeneity test for odds of conversion of *Mtb* antigen immune responses among participants with *Mtb* infection; prior vs. ongoing.

^c^ A participant without *Mtb* antigen immune responses to groups of antigens (QFT, constitutive, early, LTBI, Rv2659/Rv1196) required all of the antigen immune responses within the group to be below the cut-point.

^d^ Prior *Mtb* infection included both participants with prior notified TB and participants who received preventive monotherapy.

**Table 4:** Estimated annual reversion risk during follow-up, among participants with *Mtb* antigen immune response at least once and with subsequent assessment, regardless of *Mtb* infection status.

	Estimated annual reversion risk	Participants with *Mtb* antigen immune response at least once and with subsequent assessment	
		Revert during follow-up	Total
	%	N	N
**QFT**	**0**	0	49
**7-day IGRAs**			
** PPD**	**5**	2	52
** QFT antigens**			
Any^a^	**5**	2	53
ESAT-6	**5**	2	52
CFP10	**34**	10	35
** Constitutive antigens**			
Any^a^	**60**	8	16
Rv3614	**65**	5	9
Rv3849	**91**	11	14
Rv3865	**46**	1	3
Rv3872	**76**	3	5
** Early antigens**			
Any^a^	**44**	13	39
Rv0203	**52**	8	19
Rv0642	**45**	2	6
Rv1196	**48**	13	36
** LTBI antigens**			
Any^a^	**41**	16	50
Rv2031	**78**	13	19
Rv2244	**95**	9	9
Rv1284	**93**	12	14
Rv2659	**49**	17	47
Rv2660c	**-**	0	0
** Combinations of antigens**			
Rv2659/1196	**28**	11	54
CFP10 and Rv2659/Rv1196	**55**	14	32

The annual reversion risk was significantly higher for Rv2031 (*p<0*.*05*) among participants with ongoing (non-treated) *Mtb* infection as compared with participants with prior (treated) *Mtb* infection, see Table E in S1 File.

^a^ A positive immune response to groups of antigens (QFT, constitutive, early, LTBI, Rv2659/Rv1196) required all of the antigen immune responses within the group to be above the cut-point.

The high annual conversion and reversion risks illustrate that immune responses to *Mtb* antigens increase and decrease to a considerable extent during infection, thus these immune responses are not necessarily sustained in host immunity as an individual with *Mtb* infection can have high levels of immune responses to specific *Mtb* antigens at one point in time and low levels of immune responses below a defined test cut-point at another point in time regardless of treatment. The annual conversion and reversion risks did not differ by *Mtb* infection status (Table 3 and Table E in S1 File), but among participants with ongoing *Mtb* infection, the annual conversion risk was significantly higher for constitutive antigens (p = 0.048), the early stage antigen Rv0203 (p = 0.02) the LTBI antigen Rv2031 (p = 0.02) as compared with participants with prior *Mtb* infection. In addition, the annual reversion risk was significantly higher for Rv2031 among participants with ongoing *Mtb* infection.

To summarise the general level of non-QFT antigen conversion presented in Table 3, we calculated an estimate including all conversions for all antigens or a group of antigens as a weighted average for groups of antigens. In comparison, the annual conversion risks from Table 3 are cumulative risks for antigen conversion within each antigen group. For all participants, the weighted average conversion risk for non-QFT antigens was 22% based on annual conversion risks of 6–69% for the single antigens (Table 3), the weighted average conversion risk for the antigens in each group was 29% for early stage antigens, 13% for constitutive antigens and 24% for LTBI antigens. As different *Mtb* antigens are expressed during different stages of infection, we anticipated more conversion (change in immune response over time) among participants with early stage antigen conversion. However, participants with and without early stage antigen immune responses at enrolment had similar levels of subsequent average conversion risk for constitutive (23% vs. 25%) and LTBI antigens (19% vs. 10%). When including only participants with QFT conversion during follow-up (N = 3) in the analysis, the weighted average conversion risk for non-QFT antigens was 40% as compared to 22% for all participants, however numbers are small.

### Immunity and risk of subsequent TB

**Fig 2:**
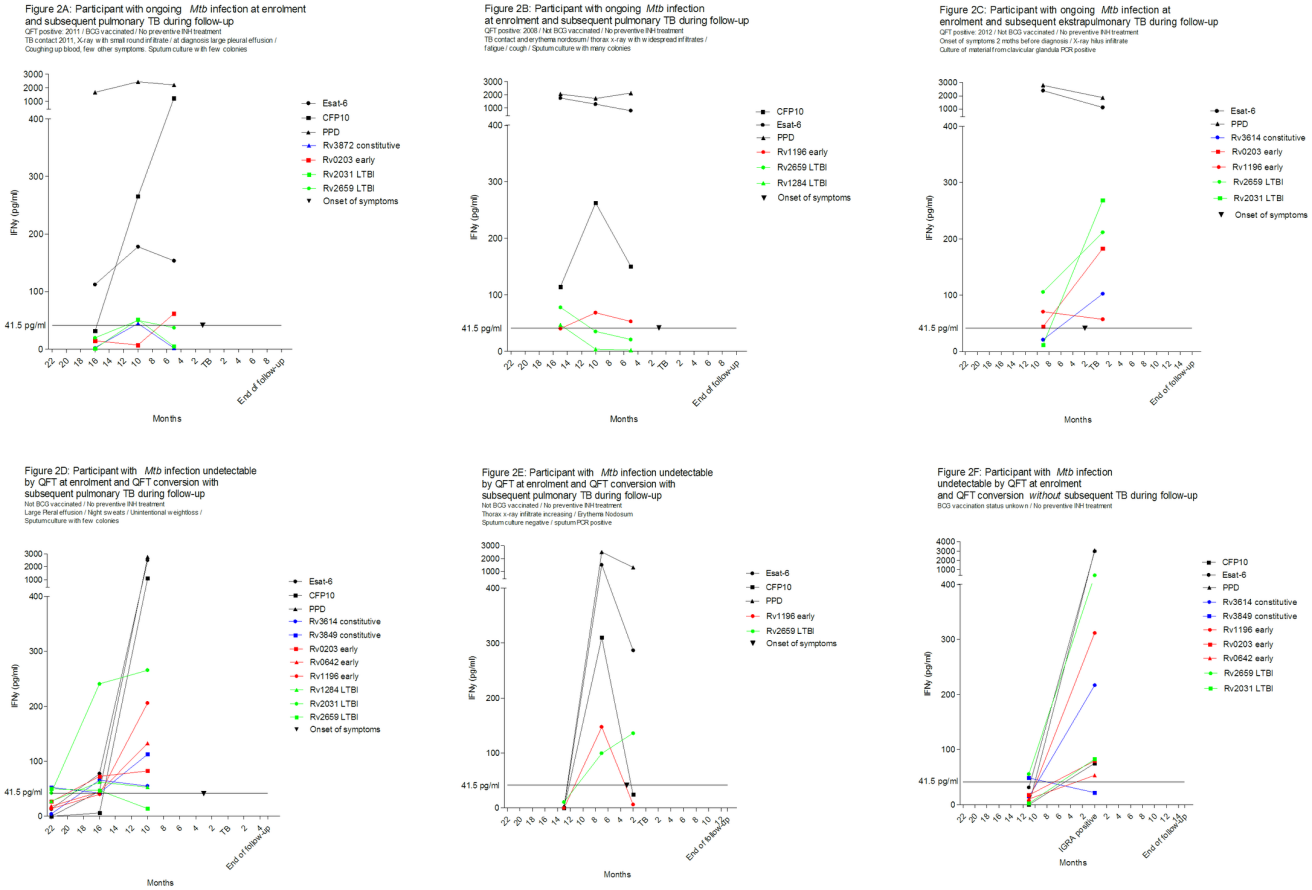
A-F. Changes in immune response to *Mtb* antigens as infection occurs and just before disease develops. Immune responses to *Mtb* antigens are shown in pg/ml after background subtraction by month of follow-up before and after TB notification or QFT conversion. Information on five participants with subsequent TB during follow-up and one participant with QFT conversion during follow-up are presented. A-C) Participants with ongoing *Mtb* infection at enrolment and subsequent TB during follow-up. D-E) Participants with *Mtb* infection undetectable by QFT at enrolment and subsequent TB during follow-up. F) One participant with *Mtb* infection undetectable by QFT at enrolment and with QFT conversion during follow-up. Information on symptom onset, clinical assessment at TB diagnosis and timing of TB diagnosis is provided.

During a total of 132 person-years, 5 (8%) participants were notified with TB, corresponding to an average TB incidence of 3,788/100,000 participants/year. Fig 2A–2E present changes in the immune response to *Mtb* antigens as infection occurs and just before disease develops in five participants with subsequent TB and one participant with QFT conversion during follow-up. The five participants were either QFT-positive at enrolment or became QFT-positive during the study period. All five had an immune response to the early stage antigen Rv1196 or LTBI antigen Rv2659. Four out of five experienced an increase in CFP10 response prior to TB development. Overall, the average conversion risk for non-QFT antigens among participants with subsequent TB was 27% vs. 21% for participants without subsequent TB, reflecting no difference in non-QFT antigen conversion by subsequent TB.

For non-QFT antigens, neither having an immune response to *Mtb* antigens upon enrolment nor a history of conversion or reversion during follow-up was found to be associated with risk of subsequent TB (Table 5). A history of QFT or ESAT-6 conversion was associated with an increased risk of TB, while a history of CFP10 conversion was not. However, a history of a substantial CFP10 response increase of ≥103.4 pg/ml was associated with a 10-fold increased risk of TB (Table 5, Fig 2A–2E). In comparison, this association was not found for the non-QFT antigens Rv1196 or Rv2659. We further investigated the timing of QFT conversion relative to the timing of subsequent TB. Three participants had QFT conversion during follow-up (Fig 2D–2F), and using this to determine the approximate time of *Mtb* infection [25], the time from infection to TB was 7 and 10 months for two participants, while no TB was observed in one participant during 21 months of follow-up (Fig C in S1 File). Similarly, among participants with a substantial CFP10 increase during follow-up (N = 21), we observed TB in four participants 7, 10, 10 and 10 months following CFP10 increase, while no TB was observed in 17 participants until end of follow-up (Fig D in S1 File).

**Table 5:** Crude Hazard ratios (HRs) for subsequent TB (N = 5) by *Mtb* antigen immune responses at enrolment, history of conversion and history of reversion during follow-up among 60 participants assessed more than once.

	Participants with *Mtb* antigen immune response at enrolment				History of conversion during follow-up				History of reversion during follow-up			
	All	TB			All	TB			All	TB		
	%	% (N)	HR (95%CI)	P	%^a^	% (N)	HR (95%CI)	P	%^a^	% (N)	HR (95%CI)	P
**QFT**	80	60 (3^f^)	0.35 (0.05–2.53)	*0*.*30*	3	40 (2)	**16.14 (1.42–183.00)**	***0*.*03***	0	0 (0)	-	-
**7-day IGRAs**												
**PPD**	80	60 (3)	0.38 (0.05–2.85)	*0*.*35*	11	40 (2)	5.12(0.64–41.20)	*0*.*13*	3	0 (0)	-	-
**QFT antigens**												
Any	80	60 (3)	0.35 (0.05–2.53)	*0*.*30*	10	40 (2)	**9.59 (1.29–71.33)**	***0*.*03***	3	0 (0)	-	-
Esat-6	78	60 (3)	0.37 (0.05–2.68)	*0*.*33*	12	40 (2)	**9.59 (1.29–71.33)**	***0*.*03***	3	0 (0)	-	-
** ***Increase*^b^	-	-	-	-	-	40 (2)	1.60 (0.24–10.74)	*0*.*63*	-	-	-	-
CFP10	45	20 (1)	0.29 (0.03–2.87)	*0*.*29*	18	60 (3)	4.17 (0.64–27.02)	*0*.*13*	14	20 (1)	2.35 (0.24–23.33)	*0*.*47*
** ***Increase*^c^	-	-	-	-	-	80 (4)	**10.06 (1.05–96.25)**	***<0*.*05***	-	-	-	-
** Constitutive Ags**												
Any	17	20 (1)	1.19 (0.13–11.1)	*0*.*88*	17	40 (2)	2.64 (0.41–17.07)	*0*.*31*	11	20 (1)	2.22 (0.21–23.89)	*0*.*51*
Rv3614	11	0 (0)	-	-	14	40 (2)	2.63 (0.42–16.27)	*0*.*30*	8	0 (0)	-	-
Rv3849	13	20 (1)	1.53 (0.17–13.99)	*0*.*71*	13	0 (0)	-	-	17	0 (0)	-	-
Rv3865	3	0 (0)	-	-	4	0 (0)	-	-	2	0 (0)	-	-
Rv3872	2	0 (0)	-	-	8	20 (1)	4.64 (0.40–54.21)	*0*.*22*	3	20 (1)	-	-
** Early Ags**												
Any	40	20 (1)	0.49 (0.05–4.63)	*0*.*53*	30	60 (3)	6.56 (0.71–60.13)	*0*.*10*	19	20 (1)	1.52 (0.16–14.66)	*0*.*72*
Rv0203	22	20 (1)	1.34 (0.13–13.5)	*0*.*80*	17	40 (2)	4.15 (0.55–30.98)	*0*.*17*	12	0 (0)	-	-
Rv0642	5	0 (0)	-	-	11	20 (1)	3.55 (0.32–29.44)	*0*.*30*	3	20 (1)	-	-
Rv1196	35	20 (1)	0.58 (0.06–5.43)	*0*.*64*	33	60 (3)	2.37 (0.39–14.27)	*0*.*34*	19	20 (1)	1.52 (0.16–14.66)	*0*.*72*
** ***Increase*^d^	-	-	-	-	-	40 (2)	1.56 (0.25–9.87)	*0*.*64*	-	-	-	-
** LTBI Ags**												
Any	55	60 (3)	1.22 (0.20–7.35)	*0*.*83*	41	40 (2)	0.88 (0.14–5.45)	*0*.*89*	23	40 (2)	2.81 (0.42–18.83)	*0*.*29*
Rv2031	20	20 (1)	0.88 (0.10–8.04)	*0*.*91*	25	40 (2)	2.11 (0.32–13.77)	*0*.*44*	18	40 (2)	3.15 (0.49–20.38)	*0*.*23*
Rv2244	6	0 (0)	-	-	12	0 (0)	-	-	13	0 (0)	-	-
Rv1284	15	20 (1)	1.76 (0.19–16.43)	*0*.*62*	12	20 (1)	1.62 (0.17–15.75)	*0*.*68*	17	20 (1)	1.09 (0.12–11.03)	*0*.*94*
Rv2659	43	60 (3)	2.67 (0.39–14.28)	*0*.*35*	50	40 (2)	0.45 (0.06–3.10)	*0*.*42*	24	40 (2)	2.34 (0.32–16.82)	*0*.*40*
** ***Increase*^e^	-	-	-	-	-	20 (1)	0.77 (0.08–7.16)	*0*.*82*	-	-	-	-
**Combinations of antigens**												
Rv2659/Rv1196	55	60 (3)	1.47 (0.24–9.19)	*0*.*68*	44	40 (2)	0.64 (0.10–4.01)	*0*.*63*	16	20 (1)	1.61 (0.16–16.32)	*0*.*69*
CFP10 and Rv2659/Rv1196	31	20 (1)	0.64 (0.06–6.84)	*0*.*71*	30	60 (3)	2.51 (0.39–16.31)	*0*.*34*	19	40 (2)	5.53 (0.67–46.46)	*0*.*11*

HRs relate to the risk of TB during follow-up with participants without detectable *Mtb* antigen immune responses as reference.

Adjustment for *Mtb* infection status did not alter the results except for the ESAT-6 conversion association. After adjustment ESAT-6 conversion was not significantly associated with risk of subsequent TB although the HR only decreased marginally HR: 9.09 (0.64–129.28), *p = 0*.*10*, see Table F in S1 File.

CI: Confidence interval. Ags: Antigens

^a^ % person years: Proportion of exposure time in person-years during a total of 96.16 person years of follow-up

^b^ ESAT-6 increase > 587 pg/ml.

^c^ CFP10 increase > 103 pg/ml.

^d^ Rv1196 increase > 97 pg/ml.

^e^ Rv2659 increase > 126 pg/ml

^f^ In Fig 2A–2F we present immune responses and clinical information (including information on treatment) for each of the participants who develop TB during follow-up and the three participants who have QFT conversion during follow-up.

## Discussion

In this study, we found that immunity to *Mtb* infection, measured by immune responses to *Mtb* antigens representing different stages of *Mtb* infection, revealed considerable fluctuation in host immunity with high conversion and reversion. Participants with ongoing *Mtb* infection at enrolment experienced significantly higher conversion as compared with participants with prior infection. High non-QFT antigen conversion was also observed at the time of infection defined by QFT conversion. Fluctuations in immune responses to the studied non-QFT antigens were not associated with subsequent TB.

The studied non-QFT antigens were *a priori* considered to be promising novel TB antigen candidates and were believed to be expressed during different stages of *Mtb* infection [26–38]. In this study, we provided the first comprehensive longitudinal data in a real-life setting for these antigens and the first human data for Rv0642, Rv2244, Rv3614, and Rv3649. Currently, only Rv2031 and Rv2659 have been evaluated longitudinally in humans [39,40], while Rv2660 and Rv1196 are present in two novel TB vaccines in clinical trial [7,41]. Our study documented that levels of conversion for non-QFT antigens are high during all stages of *Mtb* infection. The same was found for reversion, indicating that reversion may be a part of the natural fluctuations within the host immune response during *Mtb* infection. This is supported by the finding that reversion was not associated with decreased risk of subsequent TB and therefore not as proposed an indicator of an ability to contain infection [25,42].

Our findings suggest that the *Mtb* antigen repertoire exposed to the immune system during natural infection is very versatile and dynamic, and that the studied *Mtb* antigens are expressed during all infection stages. None of the non-QFT *Mtb* antigens were associated with infection stage or disease. For Rv0203, Rv2031, and any early stage antigen, conversion of immune responses were associated with ongoing *Mtb* infection, however the risk of reversion of immune responses was high for the same antigens. This implies that longitudinal and stage-specific evaluation of new vaccine and diagnostic *Mtb* antigens is necessary and supports the use of multiple antigens in novel TB vaccines.

Longitudinal studies on changes in immune responses to *Mtb* antigens are few and not conclusive. Only immune responses towards the non-QFT antigens Rv2659 and Rv2031 have been evaluated longitudinally among humans [39,40]. IFNy responses to Rv2659 and Rv2031 have been reported to increase after one and/or 40 weeks of isoniazid treatment (N = 60), and increase in Rv2031 IFNy levels among TB contacts (non-treated) as well as TB patients (during treatment) was reported during 12 months in a study from Ethiopia, however after 12 months the IFNy levels were comparable to initial IFNy levels among healthy controls from a TB-endemic region (N = 363) [39,40]. These studies only evaluated median values of IFNy responses and neither prevalence nor conversion/reversion. In the present study, we find a very high level of reversion for Rv2031 of 78%, which might make cross-sectional assessment of immune responses to Rv2031 and median IFNy levels difficult to interpret.

The finding that a substantial CFP10 response increase was associated with a 10-fold risk of subsequent TB has, to our knowledge, not previously been described in humans. However, the hypothesis emerged after data analysis and is based on small numbers which should be taken into account. Lin et al observed a significantly higher production of IFNy in response to CFP10 in peripheral blood mononuclear cells six weeks after *Mtb* infection in non-human primates (NHP) who developed TB compared with those who did not [43]. Thus, the role of CFP10 increase may be a target for examination in other independent studies, and if the above findings are confirmed, CFP10 response increase might be a potential marker for when and among whom preventive TB treatment should be initiated.

Based on the above evaluation of antigen dynamics, we cautiously suggest four characteristics of host immunity to *Mtb* infection. *Mtb* antigens exposed to the host during natural infection may be most potent at the time of infection defined by QFT conversion, reflecting an abrupt primary activation of the immune system at establishment of infection with bacterial replication affecting the host response. Participants with ongoing *Mtb* infection have a significantly higher level of antigen conversion, which might reflect a higher degree of bacterial replication during natural *Mtb* infection when left untreated, leading to the presumption that these individuals might be more susceptible to TB risk factors with suppression of cellular immunity. The substantial fluctuations observed in immune responses to non-QFT antigens suggest that host immunity among individuals with prior and ongoing *Mtb* infection is of a less dormant character than anticipated, a finding also described in NHP [44]. Finally, preceding progression from *Mtb* infection to TB, there is an alteration in immune activity, which in this study is made evident by an increase in CFP10 response. These findings contribute with important insight into the dynamics of the natural human immunity during *Mtb* infection in the absence of an animal model with the ability to capture the full spectrum of human immune characteristics of infection and disease [4].

This study has several strengths; it is unique by being a follow-up study in a setting with high TB transmission, among largely BCG-unvaccinated and NTM-unexposed individuals selected by *Mtb* infection stage. Stratified random sampling optimised the comparison of *Mtb* infection stages; however, small numbers limited the statistical power. Several features of the design minimised information bias. Information was obtained from registers, medical records and assessment at enrolment. Assessment of QFT, QFT antigens (ESAT-6, CFP10) and PPD alongside with novel non-QFT antigens contributed as additional assay controls, which allowed for an evaluation of the internal validity in the absence of a commercial assay. The definition of a positive immune response used in this study was conservative [16]. Furthermore, the observed reversions were substantial and found not to be caused by responses decreasing to levels just below the cut-point. Although the estimated levels of immune responses, conversion and reversion are naturally sensitive to the level of the cut-point, their estimated associations with *Mtb* infection stages and subsequent TB most likely are not. IFNy as single cytokine readout used to define immune responses to *Mtb* antigens is widely discussed [3]. IFNy is a robust cytokine essential in the adaptive immune response towards TB [45,46]. Furthermore, IFNy is used as a readout in standardised interferon gamma release assays (IGRAs) [14,21] and in most of the existing *Mtb* antigen research. As a supplement, an explorative multiplex assay including numerous cytokines could have expanded the evaluation [46]. However, to date, no single cytokine or pattern of cytokines have been shown to be superior, hence we found IFNy to be a suitable readout in a real-life setting with few laboratory resources [6,12,25,47–52]. Based on the above, we find it unlikely that the observed findings can be ascribed to bias.

## Conclusion

In conclusion, we found that human immunity to natural *Mtb* infection evaluated by measured immune responses to *Mtb* antigens is very versatile and shows high levels of conversion and reversion. Our findings suggest that substantial conversion and reversion of immune responses are part of the dynamics of natural *Mtb* infection, but not associated with subsequent TB development. The fluctuations in human immunity to *Mtb* antigens during infection may necessitate longitudinal assessment in the search of TB vaccine candidate antigens to account for temporary conversions and reversions.

## Supporting information

S1 File The S1 File includes the following supporting information listed in the order as referenced in the manuscript. **Table A.** Information on antigens and ratio cut-points used in definition of a positive immune response in the analyses for each *Mtb* antigen.
**Table B.** Further categorisation of year of QFT testing or TB/preventive treatment among participants at enrolment by *Mtb* infection stage among 65 young adults in East Greenland.
**Table C.** Characteristics for participants and non-participants and participation rate and OR for participation according to characteristics at enrolment.
**Fig A.** Distribution of number of immune responses to non-QFT antigens per participant by *Mtb* infection stage at enrolment.
**Table D.** Prevalence of immune responses and median IFNy response among participants with prior *Mtb* infection at enrolment by treatment status, among 65 young adults in East Greenland.
**Fig B.** Level of IFNy among reverts from first to second assessment (immune responses to 35 antigens in 19 participants).
**Table E.** Estimated annual reversion risk during follow-up, among participants with *Mtb* antigen immune response at least once and with subsequent assessment.
**Table F.** Adjusted Hazard ratios (HRs) for subsequent TB (N = 5) by *Mtb* antigen immune response at enrolment, history of conversion and history of reversion during follow-up. Adjusted for *Mtb* infection status.
**Fig C.** Percentage with subsequent TB among individuals with QFT conversion during follow-up by months since QFT conversion.
**Fig D.** Percentage with subsequent TB among individuals with a substantial CFP10 increase (≥103.39 pg/ml) during follow-up by months since CFP10 increase.
(PDF)
