# Supplementary material for: The dynamics of immune responses to Mycobacterium tuberculosis during different stages of natural infection: A longitudinal study among Greenlanders
Source: PLoS One. 2017 Jun 1;12(6):e0177906. doi: 10.1371/journal.pone.0177906 (PMC5453477; doi:10.1371/journal.pone.0177906)
Supplement: S1 File — Table A. Information on antigens and ratio cut-points used in definition of a positive immune response in the analyses for each Mtb antigen. Table B. Further categorisation of year of QFT testing or TB/preventive treatment among participants at enrolment by Mtb infection stage among 65 young adults in East Greenland. Table C. Characteristics for participants and non-participants and participation rate and OR for participation according to characteristics at enrolment. Fig A. Distribution of number of immune responses to non-QFT antigens per participant by Mtb infection stage at enrolment. Table D. Prevalence of immune responses and median IFNy response among participants with prior Mtb infection at enrolment by treatment status, among 65 young adults in East Greenland. Fig B. Level of IFNy among reverts from first to second assessment (immune responses to 35 antigens in 19 participants). Table E. Estimated annual reversion risk during follow-up, among participants with Mtb antigen immune response at least once and with subsequent assessment. Table F. Adjusted Hazard ratios (HRs) for subsequent TB (N = 5) by Mtb antigen immune response at enrolment, history of conversion and history of reversion during follow-up. Adjusted for Mtb infection status. Fig C. Percentage with subsequent TB among individuals with QFT conversion during follow-up by months since QFT conversion. Fig D. Percentage with subsequent TB among individuals with a substantial CFP10 increase (≥103.39 pg/ml) during follow-up by months since CFP10 increase. (PDF) [file pone.0177906.s001.pdf]

Supporting Information

The dynamics of immune responses to *Mycobacterium tuberculosis* during different stages of natural infection: a longitudinal study among Greenlanders

Sascha Wilk Michelsen, Bolette Soborg, Lars Jorge Diaz, Soren Tetens Hoff, Else Marie Agger, Anders Koch, Ida Rosenkrands, Jan Wohlfahrt, Mads Melbye

METHODS

Table A: Information on antigens and ratio cut-points used in definition of a positive immune response in the analyses for each *Mtb* antigen

**Antigen**							**Ratio cut-points used in definition of a positive immune response in the analyses for each *Mtb* antigen**		
**Locus tag**	**Gene description[1]**	**Known as**	**Peptides**	**Length/ overlap**	**Purity**	**Origin**	**Specificity**	**Sensitivity**	**Ratio cut-point**
-	-	PPD	-	-	-	SSI, Denmark	95%	76%	79.84
**QFT antigens**									
Rv3875	ESAT-6 protein EsxA	ESAT-6	1-7	25/10	>90%	Genecust, Luxemburg	95%	90%	35.87
Rv3874	EAST-6-like protein EsxB	CFP10	1-6	25/10	>90%	Genecust, Luxemburg	95%	98%	10.59
**Constitutive**									
Rv3614*	ESX-1 secretion-associated protein EspD	Rv3614c snm10	1-17	20/10	>80%	Genecust, Luxemburg	95%	56%	21.98
Rv3849*	ESX-1 transcriptional regulator EspR	-	1-12	20/10	>80%	Genecust, Luxemburg	95%	55%	8.94
Rv3865	ESX-1 secretion-associated protein EspF	-	1-9	20/10	>80%	Genecust, Luxemburg	95%	80%	4.22
Rv3872	PE family protein PE35	-	1-9	20/10	>80%	Genecust, Luxemburg	95%	78%	4.26
**Early**									
Rv0203	Hypothetical protein	-	1-13	20/10	>80%	Genecust, Luxemburg	95%	96%	24.29
Rv0642a*	Hydroxymycolate synthase MmaA4	Rv0642c	1-14	20/10	>80%	Genecust, Luxemburg	95%	58%	5.42
Rv0642b*	Hydroxymycolate synthase MmaA4	Rv0642c	15-29	20/10	>80%	Genecust, Luxemburg	95%	95%	3.13
Rv1196a	PPE family protein PPE18	mtb39a	1-19	20/10	>80%	Genecust, Luxemburg	95%	89%	9.39
Rv1196b	PPE family protein PPE18	mtb39a	20-33	20/10	>80%	Genecust, Luxemburg	95%	94%	14.73
**LTBI**									
Rv2031	Alpha-crystalin, HspX	Rv2031c acr	1-13	20/10	>80%	Genecust, Luxemburg	95%	95%	6.05
Rv2244*	Meromycolate extension acyl carrier protein acpM	-	1-11	20/10	>80%	Genecust, Luxemburg	95%	93%	3.13
Rv1284	Beta-carbonic anhydrase canA	-	1-15	20/10	>90%	Genecust, Luxemburg	95%	90%	16.95
Rv2659a	Prophage integrase	Rv2659c	1-13	20/10	>90%	Genecust, Luxemburg	95%	82%	15.64
Rv2659b	Prophage integrase	Rv2659c	14-25	20/10	>90%	Genecust, Luxemburg	95%	74%	78.26
Rv2659c	Prophage integrase	Rv2659c	26-37	20/10	>90%	Genecust, Luxemburg	95%	73%	57.40
Rv2660c	Hypothetical protein	Rv2660c	1-7	20/10	>90%	Genscript, USA	95%	87%	8.08

*No human or murine data. References are not exhaustive

[1]Pubmed, gene database

PPD, purified protein derivate, is in the tuberculin skin test (TST) and has a substantial antigenic overlap with BCG [1–3].

ESAT-6, Rv2660c and RV1196 are in novel TB vaccines currently in clinical testing [4].

ESAT-6 and CFP10 are two of the antigens used in QFT [1,5,6].

ESAT-6, CFP10, Rv3614, Rv3865, Rv3872 and Rv3849 are region of difference 1 (RD1) antigens and all but Rv3872 are part of the ESAT-6 antigen secretion system operon (ESX-1) [6–11].

Rv3872 and Rv1196 are part of the PE and PPE families, which constitute 10% of the *Mtb* genome [10–14].

Rv0203 is proposed to be a secreted heme transporter that mediate *Mtb* heme iron uptake [15,16].

Rv2660c, Rv1284, Rv2659, Rv2031, Rv2244 are all induced by either bacterial hypoxia or starvation, are a part of the enduring hypoxia response or dormancy regulon antigens (DosR) [17–27].

RESULTS

Table B: Further categorisation of year of QFT testing or TB/preventive treatment among participants at enrolment by *Mtb* infection stage among 65 young adults in East Greenland

Characteristics at enrolment	All		*Mtb* infection Undetectable by QFT		*Mtb*-infected								All	
					All		Non-treated		Treated (Preventive treatment)		Treated (TB treatment)		Subsequent TB	
	N	(%)	N	(%)	N	(%)	N	(%)	N	(%)	N	(%)	N	(%)
**All**	65		11		54		22		12		20		5	
**Year of: prior positive QFT or TST/ TB treatment / preventive treatment**														
1996	1	(2)	-		1	(2)	0	(0)	0	(0)	1	(5)	0	(0)
2000	1	(2)	-		1	(2)	0	(0)	0	(0)	1	(5)	0	(0)
2005	2	(3)	-		2	(4)	1	(5)	0	(0)	1	(5)	0	(0)
2008	6	(9)	-		6	(11)	6	(27)	0	(0)	0	(0)	1	(20)
2009	5	(8)	-		5	(9)	5	(23)	0	(0)	0	(0)	0	(0)
2010	5	(8)	-		5	(9)	1	(5)	3	(25)	1	(5)	0	(0)
2011	26	(40)	-		26	(48)	6	(27)	9	(75)	11	(55)	1	(20)
2012	6	(9)	-		6	(15)	3	(14)	0	(0)	3	(15)	1	(20)
In TB treatment	3	(5)	-		-	-	-	-	-	-	3	(15)	-	-
None	11	(17)	11	(100)	0	(0)	0	(0)	0	(0)	0	(0)	2	(40)

Subsequent TB: tuberculosis disease notified through the National TB register at any point from study enrolment to end of follow-up.
QFT: QuantiFERON®-TB Gold test
TST: Tuberculin skin test

Table C: Characteristics for participants and non-participants and participation rate and OR for participation according to characteristics at enrolment

Characteristics at enrolment	Non-Participants	Participants	Participation rate	OR (95% CI) for participants	
	N	N	%	Unadjusted OR	P value
**All**	116	65			
**Sex**					
Women	53	36	40	1 (ref)	
Men	63	29	32	0.68 (0.37-1.25)	*0.21*
**BCG-vaccination**					
No	100	59	37	1 (ref)	
Yes	16	6	27	0.64 (0.24-1.71)	*0.37*

Of 181 eligible, 65 participants were enrolled and tested for *Mtb* antigen immune responses, after enrolment no participants were excluded. All participants had a PHA response >74.6 pg/ml IFNγ.

Fig A

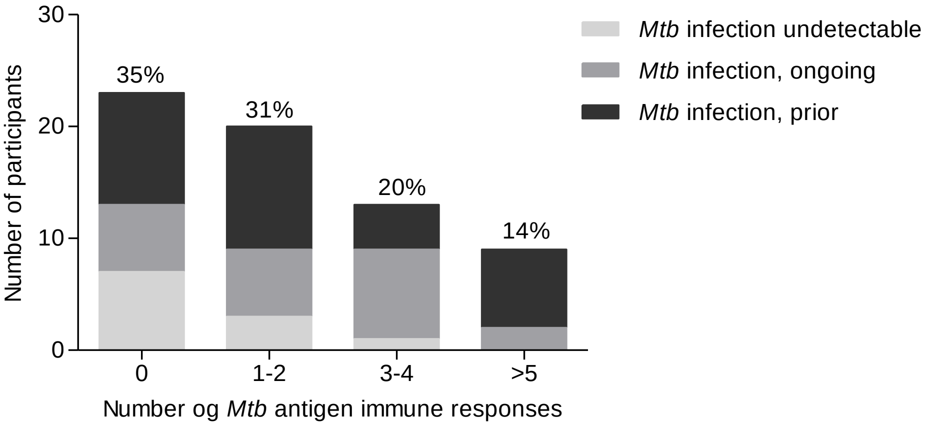

Fig A: Distribution of number of immune responses to non-QFT antigens per participant by *Mtb* infection stage at enrolment. Of 65 participants 42 (65%) participants had an immune response to at least one of the 12 non-QFT antigens. Among *Mtb*-infected participants, 70% had an immune response to at least one non-QFT antigen. 34% of participants had immune responses to three or more non-QFT antigens.

Table D: Prevalence of immune responses and median IFNy response among participants with prior *Mtb* infection at enrolment by treatment status, among 65 young adults in East Greenland

	**Prior *Mtb* infection, Treated**											
	**All**				**Treated with preventive monotherapy**				**Treated for notified TB**			
			**Median**	**(range)**			**Median**	**(range)**			**Median**	**(range)**
	**%**	**(N)**	**pg/ml**		**%**	**(N)**	**pg/ml**		**%**	**(N)**		
**All**		(32)				(12)				(20)		
**QFT**	97	(31)	157	(16-400)	92	(11)	104	(16-400)	100	(20)	203	(18-400)
**7-day IGRAs**												
**PPD**[a]	94	(30)	2177	(178-3741)	83	(10)	1397	(208-2560)	100	(20)	2463	(697-3735)
**QFT Ags**[b]												
**(any[c]/max[d])**	**97**	**(31)**	**764**	**(80-3647)**	**100**	**(12)**	**502**	**(80-3647)**	**95**	**(19)**	**764**	**(159-2807)**
ESAT-6	94	(30)	756	(80-3647)	92	(11)	485	(80-3647)	95	(19)	764	(159-2807)
CFP10	53	(17)	318	(50-784)	25	(3)	440	(50-784)	70	(14)	270	(56-765)
**Constitutive Ags**					**17**							
**(any/max)**	**17**	**(6)**	**171**	**(48-234)**		**(2)**	**174**	**(133-215)**	**20**	**(4)**	**151**	**(48-234)**
Rv3614	16	(5)	89	(42-215)	8	(1)	215	(-)	20	(4)	68	(42-209)
Rv3849[a]	16	(5)	92	(43-137)	8	(1)	137	(-)	20	(4)	73	(43-116)
Rv3865	6	(2)	90	(48-133)	8	(1)	133	(-)	5	(1)	47	(-)
Rv3872	3	(1)	234	(-)	0	(0)	-	(-)	5	(1)	234	(-)
**Early Ags**												
**(any/max)**	**44**	**(14)**	**106**	**(46-329)**	**33**	**(4)**	**94**	**(51-180)**	**50**	**(10)**	**106**	**(46-329)**
Rv0203[a]	19	(6)	85	(43-329)	8	(1)	131	(-)	25	(5)	74	(43-329)
Rv0642	3	(1)	130	(-)	0	(0)	-	(-)	5	(1)	130	(-)
Rv1196	44	(14)	87	(45-329)	33	(4)	57	(51-180)	50	(10)	106	(45-329)
**LTBI Ags**												
**(any/max)**	**60**	**(19)**	**177**	**(44-918)**	**50**	**(6)**	**58**	**(44-491)**	**65**	**(13)**	**184**	**(45-918)**
Rv2031[a]	22	(7)	184	(66-624)	17	(2)	163	(66-260)	25	(5)	184	(79-624)
Rv2244	9	(3)	57	(46-120)	0	(0)	-	(-)	15	(3)	56	(46-120)
Rv1284	22	(7)	71	(50-918)	8	(1)	50	(-)	30	(6)	92	(62-918)
Rv2659	47	(15)	62	(44-491)	33	(4)	47	(44-491)	55	(11)	71	(45-478)
Rv2660c	0	(0)	-	(-)	0	(0)	-	(-)	0	(0)	-	(-)
**Combination of Ags**												
Rv2659/Rv1196	63	(20)	90	(44-491)	58	(7)	51	(44-491)	65	(13)	124	(49-477)

[a] One donor missing one stimulation. [b] Ags: antigens. [c] Immune responses for groups of antigens (QFT, constitutive, early, LTBI, Rv2659/Rv1196) were defined as having an immune response to at least one of the antigens and [d] the IFNy response magnitude was defined by the maximum response.

Fig B

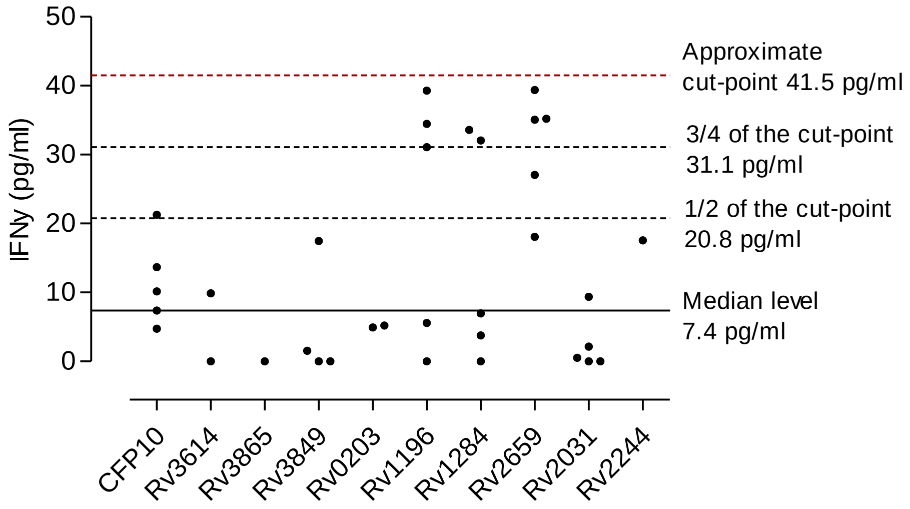

Fig B: Level of IFNy among reverts from first to second assessment (35 immune responses to 10 antigens in 19 participants). Rv1196 and Rv2659 response levels were generated from the maximum response of the included pools, which partly explains higher values for these two antigens compared to other antigens. The median IFNy level was 7.4 pg/ml. 80% the IFNy levels were less than ¾ of the cut-point level. When not considering Rv1196 and Rv2659, 97% of the IFNy levels were less that ¾ of the cut-point level.

Table E: Estimated annual reversion risk during follow-up, among participants with *Mtb* antigen immune response at least once and with subsequent assessment

Antigens	All: Estimated annual reversion risk %	All: Participants with *Mtb* antigen immune response at least once and with subsequent assessment: Revert during follow-up N	All: Total N	*Mtb* infection undetectable by QFT: Estimated annual reversion risk %	*Mtb* infection undetectable by QFT: Revert during follow-up N	*Mtb* infection undetectable by QFT: Total N	*Mtb* infection, All: Estimated annual reversion risk %	*Mtb* infection, All: Revert during follow-up N	*Mtb* infection, All: Total N	Reversion risk: *Mtb*-infection QFT un-detectable vs detectable P value[a]	*Mtb* infection, Ongoing, non-treated: Estimated annual reversion risk %	Ongoing, non-treated: Revert during follow-up N	Ongoing, non-treated: Total N	*Mtb* infection, Prior, treated[d]: Estimated annual reversion risk %	Prior, treated[d]: Revert during follow-up N	Prior, treated[d]: Total N	Rever-sion risk: on-going vs. prior P value[b]
**QFT**	**0**	0	49	**0**	0	1	**0**	0	48	-	**0**	0	19	**0**	0	29	-
**7-day IGRAs**																	
**PPD**	**5**	2	52	**0**	0	4	**5**	2	48	-	**6**	1	20	**4**	1	28	*0.79*
**QFT Antigens**																	
Any[c]	**5**	2	53	**0**	0	3	**5**	2	50	-	**6**	1	20	**4**	1	30	*0.78*
ESAT-6	**5**	2	52	**0**	0	3	**5**	2	49	-	**6**	1	20	**4**	1	29	*0.77*
CFP10	**34**	10	35	**100**	1	1	**32**	9	34	*1.00*	**8**	1	15	**48**	8	19	*0.06*
**Constitutive antigens**																	
Any[c]	**60**	8	16	**58**	2	4	**61**	6	12	*0.93*	**69**	3	20	**55**	3	6	*0.64*
Rv3614	**65**	5	9	**75**	1	2	**63**	4	7	*0.77*	**58**	1	2	**65**	3	5	*0.87*
Rv3849	**91**	11	14	**83**	3	4	**94**	8	10	*0.53*	**84**	3	5	**99**	5	5	*0.29*
Rv3865	**46**	1	3	-	0	0	**46**	1	3	-	**0**	0	1	**59**	1	2	-
Rv3872	**76**	3	5	-	0	0	**76**	3	5	-	**89**	2	3	**58**	1	2	*0.55*
**Early antigens**																	
Any[c]	**44**	13	39	**75**	1	2	**42**	12	37	*0.38*	**38**	5	18	**47**	7	19	*0.63*
Rv0203	**52**	8	19	**0**	0	1	**53**	8	18	-	**73**	7	12	**17**	1	6	*0.07*
Rv0642	**45**	2	6	**0**	0	1	**51**	2	5	-	**45**	1	3	**58**	1	2	*0.80*
Rv1196	**48**	13	36	**100**	1	1	**45**	12	35	*1.00*	**44**	5	16	**47**	7	19	*0.88*
**LTBI antigens**																	
Any[c]	**41**	16	50	**18**	1	8	**45**	15	42	*0.29*	**54**	8	19	**38**	7	23	*0.34*
Rv2031	**78**	13	19	**79**	1	1	**78**	12	18	*0.97*	**94**	8	9	**55**	4	9	***<0.05***
Rv2244	**95**	9	9	-	0	0	**95**	9	9	-	**91**	2	2	**96**	7	7	*0.72*
Rv1284	**93**	12	14	**75**	1	2	**95**	11	12	*0.48*	**100**	4	4	**90**	7	8	-
Rv2659	**49**	17	47	**18**	1	8	**55**	16	39	*0.18*	**63**	8	18	**49**	8	21	*0.43*
Rv2660c	-	0	0	-	0	0	-	0	0	-	-	0	0	-	0	0	-
**Combinations of antigens[c]**																	
Rv2659/ Rv1196	**28**	11	54	**18**	1	8	**29**	10	46	*0.60*	**29**	4	20	**30**	6	26	*0.92*
CFP10 and Rv2659/ Rv1196	**55**	14	32	**100**	1	1	**52**	13	31	*1.00*	**38**	4	14	**63**	9	17	*0.22*

The table presents three columns per group (e.g. all, *Mtb* infection undetectable, *Mtb* infection). The right column is the total number of participants with *Mtb* antigen immune responses at either the first or the second assessment, thus have the possibility to revert (e.g. for Rv0203, 19 participants with *Mtb* antigen immune responses had the possibility to revert). The middle column shows the number of participants who revert during follow-up (e.g. for Rv0203, 8 of 19 participants revert during follow-up), and the left column shows the estimated annual reversion risk (e.g. for Rv0203 the annual conversion risk is 52%).

[a] P values relate to homogeneity test for odds of *Mtb* antigen reversion among all participants by *Mtb* infection status (udetectable/detectable *Mtb* infection). [b] P values relate to homogeneity test for odds of *Mtb* antigen reversion among participants with *Mtb* infection by treatment status. [c] For groups of antigens (QFT antigens, constitutive, early, LTBI and Rv2659/1196) immune responses to at least one of the antigens defined having a positive immune response, while a negative immune response required all of the antigen immune responses within the group to be below the cut-point. [d] Treated participants were defined by both prior LTBI and prior TB treatment.

Table F. Adjusted Hazard ratios (HRs) for subsequent TB (N=5) by *Mtb* antigen immune response at enrolment, history of conversion and history of reversion during follow-up. Adjusted for *Mtb* infection status

	Participants with *Mtb* antigen immune response at enrolment and risk of subsequent TB		History of conversion during follow-up and risk of subsequent TB		History of reversion during follow-up and risk of subsequent TB	
**Antigens**	**HR (95%CI)**	**P value**	**HR (95%CI)**	**P value**	**HR (95%CI)**	**P value**
**QFT**	-	-	-	-	-	-
**7-day IGRAs**						
**PPD**	-	-	3.77 (0.30-47.75)	*0.31*	-	-
**QFT antigens**						
Any	-	-	9.09 (0.64-129.28)	*0.10*	-	-
Esat-6	-	-	9.09 (0.64-129.28)	*0.10*	-	-
CFP10	0.38 (0.03-4.40)	*0.44*	3.86 (0.57-26.07)	*0.17*	3.28 (0.30-26.44)	*0.33*
**Constitutive antigens**						
Any	1.03 (0.11-9.69)	*0.98*	2.19 (0.33-14.42)	*0.42*	1.98 (0.18-21.23)	*0.57*
Rv3614	-	-	2.01 (0.29-13.83)	*0.48*	-	-
Rv3849	1.20 (0.13-11.53)	*0.87*	-	-	-	-
Rv3865	-	-	-	-	-	-
Rv3872	-	-	-	-	-	-
**Early antigens**						
Any	0.69 (0.06-7.88)	*0.77*	6.97 (0.73-66.39)	*0.09*	1.90 (0.19-19.52)	*0.59*
Rv0203	2.05 (0.17-24.92)	*0.57*	3.48 (0.46-26.09)	*0.23*	-	-
Rv0642	-	-	2.45 (0.19-30.86)	*0.49*	-	-
Rv1196	0.83 (0.07-9.26)	*0.88*	2.47 (0.41-15.05)	*0.33*	1.90 (0.19-19.52)	*0.59*
**LTBI antigens**						
Any	1.28 (0.21-7.81)	*0.79*	0.86 (0.14-5.43)	*0.87*	3.96 (0.48-32.73)	*0.20*
Rv2031	1.08 (0.12-10.20)	*0.94*	2.68 (0.37-19.34)	*0.34*	3.86 (0.55-26.99)	*0.17*
Rv2244	-	-	-	-	-	-
Rv1284	2.69 (0.24-30.49)	*0.42*	1.06 (0.10-10.92)	*0.96*	1.15 (0.11-12.05)	*0.91*
Rv2659	2.08 (0.34-12.59)	*0.42*	0.50 (0.07-3.62)	*0.50*	3.27 (0.37-29.01)	*0.29*
**Combinations**						
Rv2659/Rv1196	1.40 (0.23-8.46)	*0.72*	0.71 (0.11-4.56)	*0.72*	1.77 (0.17-18.19)	*0.63*
CFP10 and Rv2659/Rv1196	0.83 (0.07-10.32)	*0.89*	2.45 (0.37-16.13)	*0.35*	5.95 (0.72-49.06)	*0.10*

HRs relate to the risk of TB during follow-up.

Fig C

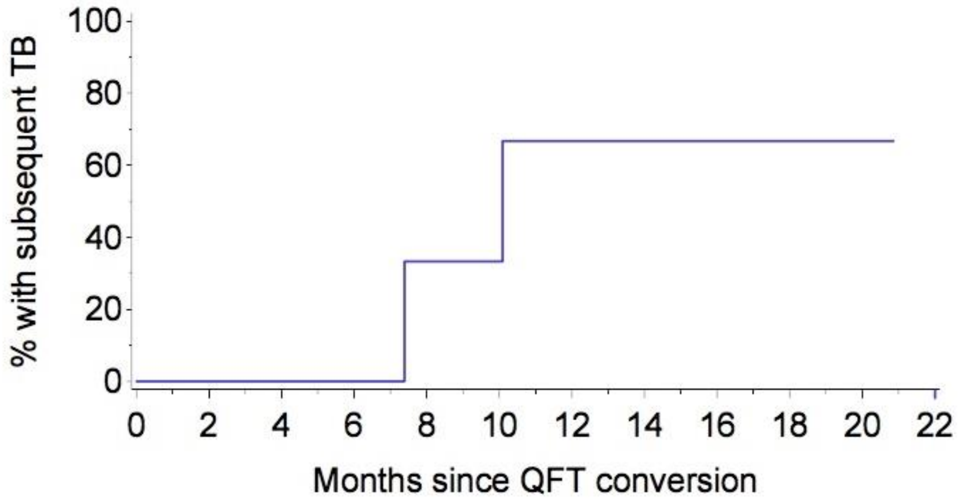

Fig C: Percentage with subsequent TB among individuals with QFT conversion during follow-up by months since QFT conversion (estimated by the Kaplan Meier estimator). Three participants had QFT conversion during follow-up, two progress to subsequent TB. The time from infection to TB was 7 and 10 months for two participants while no TB was observed in one participant during 21 months of follow-up.

Fig D

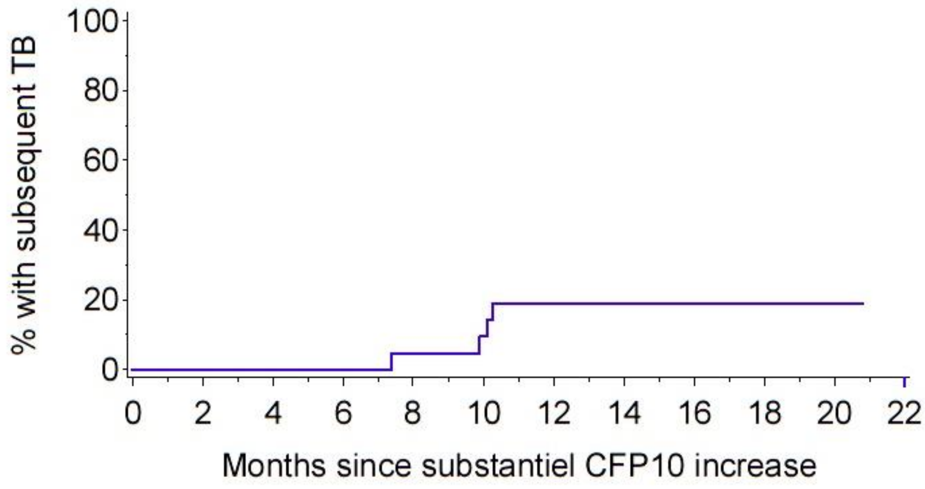

Fig D: Percentage with subsequent TB among individuals with a substantial CFP10 increase (≥103.39 pg/ml) during follow-up by months since CFP10 increase (estimated by the Kaplan Meier estimator). A substantial CFP10 increase was observed for 21 participants had during follow-up, four progress to subsequent TB. The time from CFP10 increase to TB was 7, 10, 10 and 10 months for four participants while no TB was observed in 17 participants until end of follow-up

References

1. Pai M, Riley LW, Colford JM. Interferon-gamma assays in the immunodiagnosis of tuberculosis: a systematic review. Lancet Infect Dis. 2004;4: 761–76. doi:10.1016/S1473-3099(04)01206-X
2. Andrews JR, Hatherill M, Mahomed H, Hanekom W a., Campo M, Hawn TR, et al. The Dynamics of QuantiFERON-TB Gold In-Tube Conversion and Reversion in a Cohort of South African Adolescents. Am J Respir Crit Care Med. 2015;191: 584–591. doi:10.1164/rccm.201409-1704OC
3. Huebner RE, Maybelle FS, Bass JBJ. The tuberculin skin test. Clin Infect Dis. 1993;17: 968–75. doi:10.1002/path.1711550102
4. Hawn TR, Day TA, Scriba TJ, Hatherill M, Hanekom WA, Evans TG, et al. Tuberculosis Vaccines and Prevention of Infection. Microbiol Mol Biol Rev. 2014;78: 650–671. doi:10.1128/MMBR.00021-14
5. Andersen P, Munk ME, Pollock JM, Doherty TM. Specific immune-based diagnosis of tuberculosis. Lancet. 2000;356: 1099–1104. Available: http://www.ncbi.nlm.nih.gov/pubmed/11009160
6. Brodin P, Majlessi L, Marsollier L, De Jonge MI, Bottai D, Demangel C, et al. Dissection of ESAT-6 system 1 of Mycobacterium tuberculosis and impact on immunogenicity and virulence. Infect Immun. 2006;74: 88–98. doi:10.1128/IAI.74.1.88-98.2006
7. Raghavan S, Manzanillo P, Chan K, Dovey C, Cox JS. Secreted transcription factor controls Mycobacterium tuberculosis virulence. Nature. 2008;454: 717–721. doi:10.1038/nature07219
8. Chen JM, Boy-Röttger S, Dhar N, Sweeney N, Buxton RS, Pojer F, et al. EspD is critical for the virulence-mediating ESX-1 secretion system in Mycobacterium tuberculosis. J Bacteriol. 2012;194: 884–893. doi:10.1128/JB.06417-11
9. Bahk YY, Kim SA, Kim J-S, Euh H-J, Bai G-H, Cho S-N, et al. Antigens secreted from Mycobacterium tuberculosis: identification by proteomics approach and test for diagnostic marker. Proteomics. 2004;4: 3299–3307. doi:10.1002/pmic.200400980
10. Mukherjee P, Dutta M, Datta P, Dasgupta a., Pradhan R, Pradhan M, et al. The RD1-encoded antigen Rv3872 of Mycobacterium tuberculosis as a potential candidate for serodiagnosis of tuberculosis. Clin Microbiol Infect. European Society of Clinical Infectious Diseases; 2007;13: 146–152. doi:10.1111/j.1469-0691.2006.01660.x
11. Tiwari B, Soory A, Raghunand TR. An immunomodulatory role for the Mycobacterium tuberculosis region of difference 1 locus proteins PE35 (Rv3872) and PPE68 (Rv3873). FEBS J. 2014;35: 1556–1570. doi:10.1111/febs.12723
12. Bhat KH, Ahmed A, Kumar S, Sharma P, Mukhopadhyay S. Role of PPE18 Protein in Intracellular Survival and Pathogenicity of Mycobacterium tuberculosis in Mice. PLoS One. 2012;7: 1–9. doi:10.1371/journal.pone.0052601
13. Dillon DC, Alderson MR, Day CH, Lewinsohn DM, Coler R, Bement T, et al. Molecular characterization and human T-cell responses to a member of a novel Mycobacterium tuberculosis mtb39 gene family. Infect Immun. 1999;67: 2941–2950.
14. Nair S, Pandey AD, Mukhopadhyay S. The PPE18 protein of Mycobacterium tuberculosis inhibits NF-κB/rel-mediated proinflammatory cytokine production by upregulating and phosphorylating suppressor of cytokine signaling 3 protein. J Immunol. 2011;186: 5413–5424. doi:10.4049/jimmunol.1000773
15. Amor YB, Shashkina E, Johnson S, Bifani PJ, Kurepina N, Kreiswirth B, et al. Immunological characterization of novel secreted antigens of Mycobacterium tuberculosis. Scand J Immunol. 2005;61: 139–146. doi:10.1111/j.0300-9475.2005.01557.x

16. Owens CP. Characterization of heme ligation properties of Rv0203, a secreted heme binding protein involved in Mycobacterium tuberculosis heme uptake. Biochemistry. 2012;29: 997–1003. doi:10.1016/j.biotechadv.2011.08.021.Secreted

17. Lin PL, Dietrich J, Tan E, Abalos RM, Burgos J, Bigbee C, et al. The multistage vaccine H56 boosts the effects of BCG to protect cynomolgus macaques against active tuberculosis and reactivation of latent Mycobacterium tuberculosis infection. J Clin Invest. 2012;122: 303–314. doi:10.1172/JCI46252.ESAT-6

18. Ottenhoff THM, Kaufmann SHE. Vaccines against tuberculosis: Where are we and where do we need to go? PLoS Pathog. 2012;8: e1002607. doi:10.1371/journal.ppat.1002607

19. Michelsen SW, Soborg B, Agger EM, Diaz LJ, Hoff ST, Koch A, et al. Host immunity to Mycobacterium tuberculosis and risk of tuberculosis: a longitudinal study among Greenlanders. Vaccine. 2016;34: 5975–5983. doi:http://dx.doi.org/10.1016/j.vaccine.2016.09.047

20. Govender L, Abel B, Hughes EJ, Scriba TJ, Kagina BMN, de Kock M, et al. Higher human CD4 T cell response to novel Mycobacterium tuberculosis latency associated antigens Rv2660 and Rv2659 in latent infection compared with tuberculosis disease. Vaccine. Elsevier Ltd; 2010;29: 51–7. doi:10.1016/j.vaccine.2010.10.022

21. Gideon HP, Wilkinson K a, Rustad TR, Oni T, Guio H, Sherman DR, et al. Bioinformatic and empirical analysis of novel hypoxia-inducible targets of the human antituberculosis T cell response. J Immunol. 2012;189: 5867–76. doi:10.4049/jimmunol.1202281

22. Commandeur S, van Meijgaarden KE, Prins C, Pichugin A V, Dijkman K, van den Eeden SJF, et al. An unbiased genome-wide Mycobacterium tuberculosis gene expression approach to discover antigens targeted by human T cells expressed during pulmonary infection. J Immunol. 2013;190: 1659–71. doi:10.4049/jimmunol.1201593

23. Torres M, Garcıa-Garcia L, Cruz-Hervert P, Guio H, Carranza C, Ferreyra-Reyes L. Effect of isoniazid on antigen-specific interferon-c secretion in latent tuberculosis. Eur Respir J. 2015;45: 473–82. doi:10.1183/09031936.00123314

24. Legesse M, Ameni G, Medhin G, Mamo G, Franken KLMC, Ottenhoff THM, et al. IgA Response to ESAT-6/CFP-10 and Rv2031 Antigens Varies in Patients With Culture-Confirmed Pulmonary Tuberculosis, Healthy Mycobacterium tuberculosis-Infected and Non-Infected Individuals in a Tuberculosis Endemic Setting, Ethiopia. Scand J Immunol. 2013;78: 266–274. doi:10.1111/sji.12080

25. Sherman DR, Voskuil M, Schnappinger D, Liao R, Harrell MI, Schoolnik GK. Regulation of the Mycobacterium tuberculosis hypoxic response gene encoding alpha -crystallin. Proc Natl Acad Sci U S A. 2001;98: 7534–9. doi:10.1073/pnas.121172498

26. Belay M, Legesse M, Mihret A, Bekele Y, Ottenhoff THM, Franken KLMC, et al. Pro- and Anti-Inflammatory Cytokines against Rv2031 Are Elevated during Latent Tuberculosis: A Study in Cohorts of Tuberculosis Patients, Household Contacts and Community Controls in an Endemic Setting. PLoS One. 2015;10: e0124134. doi:10.1371/journal.pone.0124134

27. Kremer L, Nampoothiri KM, Lesjean S, Dover LG, Graham S, Betts J, et al. Biochemical Characterization of Acyl Carrier Protein (AcpM) and Malonyl-CoA:AcpM Transacylase (mtFabD), Two Major Components of Mycobacterium tuberculosis Fatty Acid Synthase II. J Biol Chem. 2001;276: 27967–27974. doi:10.1074/jbc.M103687200
